# Stable production of cyanophycinase in *Nicotiana benthamiana* and its functionality to hydrolyse cyanophycin in the murine intestine

**DOI:** 10.1111/pbi.12658

**Published:** 2016-12-18

**Authors:** Daniel Ponndorf, Sven Ehmke, Benjamin Walliser, Kerstin Thoss, Christoph Unger, Solvig Görs, Gürbüz Daş, Cornelia C. Metges, Inge Broer, Henrik Nausch

**Affiliations:** ^1^ Faculty of Agricultural and Environmental SciencesDepartment of Agrobiotechnology and Risk Assessment for Bio‐ and Gene TechnologyUniversity of RostockRostockGermany; ^2^ Leibniz Institute for Farm Animal Biology (FBN)Institute of Nutritional Physiology ‘Oskar Kellner’DummerstorfGermany; ^3^ Present address: Paraxel International GmbHKlinikum am Westend, Haus 18, SpandauerDamm 130, 14050BerlinGermany

**Keywords:** arginine, cyanophycin, cyanophycinase, dipeptide, digestion, *Nicotiana benthamiana*, protein stability

## Abstract

Food supplementation with the conditionally essential amino acid arginine (Arg) has been shown to have nutritional benefits. Degradation of cyanophycin (CGP), a peptide polymer used for nitrogen storage by cyanobacteria, requires cyanophycinase (CGPase) and results in the release of β‐aspartic acid (Asp)‐Arg dipeptides. The simultaneous production of CGP and CGPase in plants could be a convenient source of Arg dipeptides. Different variants of the *cph*B coding region from *Thermosynechococcus elongatus* BP‐1 were transiently expressed in *Nicotiana benthamiana* plants. Translation and enzyme stability were optimized to produce high amounts of active CGPase. Protein stability was increased by the translational fusion of CGPase to the green fluorescent protein (GFP) or to the transit peptide of the small subunit of RuBisCO for peptide production in the chloroplasts. Studies in mice showed that plant‐expressed CGP fed in combination with plant‐made CGPase was hydrolysed in the intestine, and high levels of ß‐Asp‐Arg dipeptides were found in plasma, demonstrating dipeptide absorption. However, the lack of an increase in Asp and Arg or its metabolite ornithine in plasma suggests that Arg from CGP was not bioavailable in this mouse group. Intestinal degradation of CGP by CGPase led to low intestinal CGP content 4 h after consumption, but after ingestion of CGP alone, high CGP concentrations remained in the large intestine; this indicated that intact CGP was transported from the small to the large intestine and that CGP was resistant to colonic microbes.

## Introduction

Arginine (Arg) is an indispensable amino acid (AA) for young mammals and birds (Wu *et al*., 2004, 2009). In addition to its function as a building block of proteins, Arg plays important roles in regulating gene expression, cell signalling, vascular development, reproduction and immunity (Bazer *et al*., 2012; Wang *et al*., 2012; Wu, 2014). Furthermore, Arg has nutritional benefits for athletes and the elderly or immune‐compromised patients, but because its concentration is relatively low in food proteins, it has been used as a supplement for therapy and as an additive in food (Sallam and Steinbuchel, 2010). Supplemental free Arg is commonly produced by fermentation (Utagawa, 2004). The oral application of Arg‐containing dipeptides may increase the uptake of Arg in the small intestine compared to Arg monomers (Matthews and Adibi, 1976; Wenzel *et al*., 2001). Currently, dipeptides are synthesized by enzymatic, chemical and combined methods (Yagasaki and Hashimoto, 2008). Overexpression of the polypeptide cyanophycin (CGP) followed by cyanophycinase (CGPase)‐mediated degradation results in β‐aspartic acid (Asp)‐Arg dipeptides, which could produce Arg (Sallam and Steinbuchel, 2009b, 2010; Sallam *et al*., 2009). Cyanophycin is synthesized by cyanobacteria and several nonphotosynthetic bacteria via nonribosomal biosynthesis by the enzyme cyanophycin synthetase (CPHA) (Allen *et al*., 1984; Simon, 1987; Simon and Weathers, 1976; Ziegler *et al*., 1998, 2002) and consists of an L‐Asp backbone linked to L‐Arg residues (Simon and Weathers, 1976). The expression of the CPHA‐encoding gene from *Thermosynechococcus elongatus* BP‐1 enables high and stable accumulation of CGP in tobacco and potato plastids (Hühns *et al*., 2008, 2009; Neumann *et al*., 2005). However, to the best of our knowledge, there are no reports on feeding CGP to animals and assessing its potential to produce Arg. CGP is highly stable and resistant to proteases (Simon and Weathers, 1976), except CGPase (Gupta and Carr, 1981). The cyanophycinase CPHB was described in cell extracts of *Anabaena 7120* (Gupta and Carr, 1981)*, Aphanocapsa 6308* (Allen *et al*., 1984) and *Synechocystis* sp. PCC 6803 (Richter *et al*., 1999). Overexpression in *E.coli* and analysis of CPHB revealed that it is a serine (Ser)‐type exopeptidase with a dimeric structure (Law *et al*., 2009; Richter *et al*., 1999), and its binding is highly specific for β‐linked aspartyl peptides. If CGP and CGPase can be co‐expressed in food plants, β‐Asp‐Arg dipeptides could become a source of dietary Arg. The degradation of CGP resulting in the release of β‐Asp‐Arg dipeptides might be achieved following two strategies: (i) accumulation of CGP in the plastid via separation of CGP (plastid) and CGPase (cytosol) leading to dipeptide formation after extraction when both components are joined and (ii) accumulation of the dipeptides in plastids by targeting cyanophycin synthetase (CPHA) and cyanophycinase (CPHB) to the chloroplast allowing dipeptide production during plant growth.

We used a transient expression system in *N. benthamiana* to determine whether plants can produce an active and stable form of CGPase that degrades CGP and whether the enzyme can be translocated to the chloroplast. In the second step, we formulated food pellets containing both, plant‐made CGP and plant‐made CGPase to investigate whether CGP is hydrolysed by CGPase and whether Arg from CGP is bioavailable in a mouse model.

## Results

### Cytosolic production and stabilization of CGPase in plants

**Figure 1:**
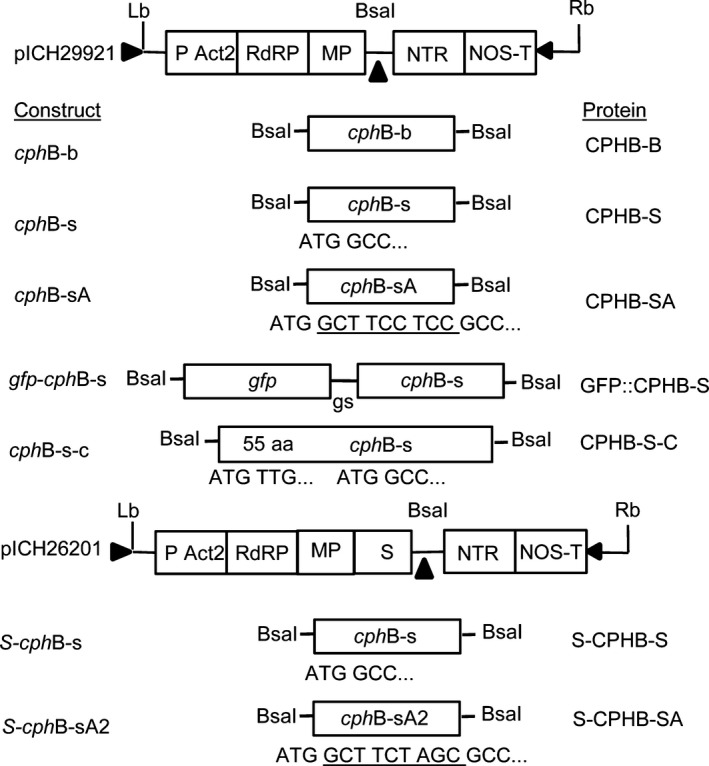
Plasmid constructs and corresponding labels of the respective protein variants: TMV‐based viral vectors (Marillonnet *et al*., 2005): pICH29912: empty vector, cphB‐b: bacterial coding region of the cphB gene, cphB‐s: cphB coding region adapted to the codon usage of *N. benthamiana*, cphB‐sA: cphB‐s with the addition of the amino acids Ala‐Ser‐Ser (A) (underlined sequence), gfp‐cphB‐s: fusion of gfp (sequence of the green fluorescent protein from pICH18711 (Marillonnet *et al*., 2005)) and cphB‐s, gs: linker, cphB‐s‐c: complete codon‐optimized coding region of cphB as described in the database; pICH26201: containing a consensus sequence of the transit peptide of small subunit of RuBisCO (S) from dicotyl plants (Klimyuk *et al*., 2004), cphB‐sA2: cphB‐s with the addition of the altered sequence of amino acids Ala‐Ser‐Ser (underlined sequence). LB and RB: left and right T‐DNA borders; P Act2: Arabidopsis actin 2 promoter; RdRP: RNA‐dependent RNA polymerase; MP: movement protein; NTR: 3ˈ untranslated region of TMV; NOS‐T: nos terminator.

**Figure 2:**
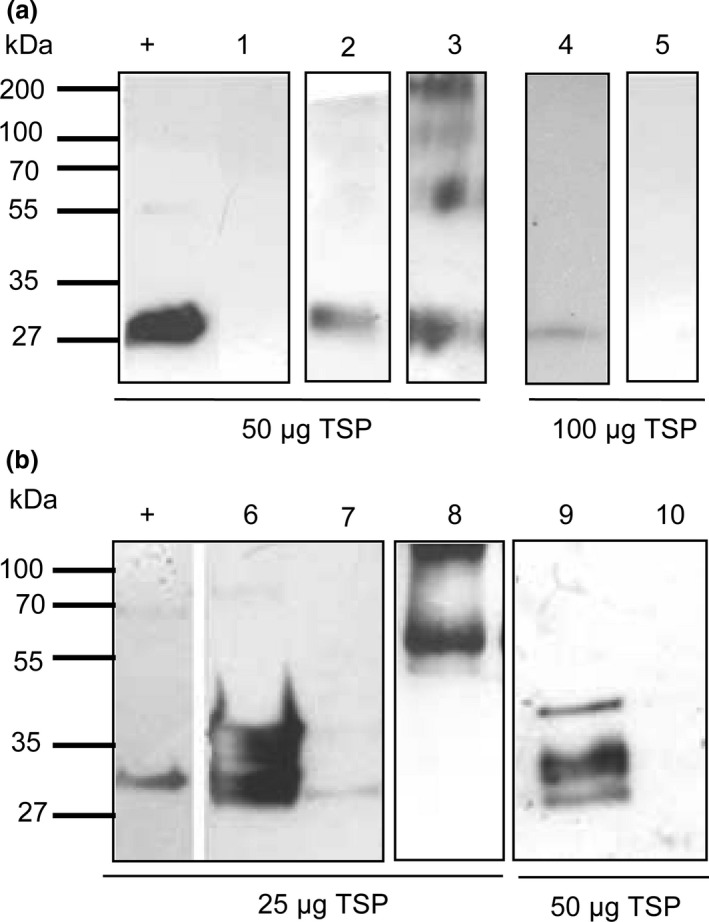
(a) Western blot analysis of 50 and 100 μg total soluble protein isolated from *N. benthamiana* leaves harvested 7 days post infiltration from one plant (dpi): + = Cyanophycinase‐positive control isolated from *E. coli*; 1: CPHB‐B; 2: CPHB‐S; 3:CPHB‐SA, 4: CPHB‐B; 5: pICH29912 = empty vector control. b: 25 and 50 μg TSP harvested 7 dpi from one plant:+ = Cyanophycinase‐positive control isolated from *E. coli* 6:S‐CPHB‐S, 7:S‐CPHB‐SA2; 8: GFP::CPHB‐S; 9: CPHB‐S‐C; 10:pICH29912 =  empty vector control. Plants were harvested 7 dpi.

A 5ˈ truncated coding region of the *cph*B_tlr2169_ gene (*cph*B‐b) (Prof. Dr. Wolfgang Lockau (W.L.) Humbold Univesity Berlin) and two codon‐optimized versions, *cph*B‐s, designed to improve the efficiency of translation (Perlak *et al*., 1991; Sharp and Li, 1987) and *cph*B‐sA, where the sequence GCT TCCTCC encoding for Alanin (Ala)‐Ser‐Ser (Fig. 1), was added to improve efficiency of translation and protein stability (Sawant *et al*., 2001) were transiently expressed in *N. benthamiana*. Total soluble protein (TSP) was assessed for the presence of CGPase using Western blot analysis (Fig. 2a). Infiltration with *cph*B‐b did not result in detection of CGPase in 50 μg TSP, but faint, not always reproducible, signals were visible at the expected size of approximately 29 kDa in 100 μg TSP. Expression of *cph*B‐s and analysis of 50 μg TSP showed faint CGPase signals, while expression of *cph*B‐sA resulted in more pronounced signals. Bands were detected not only at 29 kDa but also at 60, 130 and 200 kDa. These bands were not observed in the empty vector control and their size corresponds to potential di‐ and trimers and higher aggregates of CPHB‐SA. Additionally, we were not able to detect CGPase without the addition of protease inhibitors, suggesting its instability in the crude plant extract. Greater stability of recombinant proteins can be achieved by fusion of the protein to a stable fusion partner at the *N‐* or *C‐*terminus. One promising fusion partner is GFP which was previously used successfully to improve protein stability (Piron *et al*., 2014). Because our constructs carry a 6*Histag at the *C‐*terminus for enzyme purification, we used *N*‐terminal fusions. The fusion of *cph*B‐s to the green fluorescent protein (GFP) coding region, resulting in *gfp*‐*cph*B‐s, led to an increase in protein yield as shown in Figure 2b. An additional band was visible at approximately 100 kDa, corresponding to the calculated size of a GFP::CPHB‐S dimer.

Because *N*‐terminal fusions stabilized the protein, we analysed whether the complete CGPase protein (CPHB‐S‐C) described in the database is more stable compared to the truncated version. Therefore, we used a codon‐optimized version of the complete sequence. After infiltration with *cph*B‐s‐c, three bands were detected in 50 μg TSP at approximately 27, 29 and 35 kDa. The 35‐kDa protein corresponds to the calculated size of the complete protein. The smaller bands are potential degradation products, while the band at 29 kDa is the size of the truncated protein.

### Subcellular targeting of CPHB to the chloroplast

To determine whether CPHB‐S and CPHB‐SA can be targeted to the chloroplast *cph*B‐s was fused to the plastid leader peptide of the small subunit of RuBisCO (S) (Klimyuk *et al*., 2004) resulting in S‐*cph*B‐s. Due to sequence incompatibility between S and the sequence GCT TCC TCC of Ala‐Ser‐Ser (A) in *cph*B‐sA, it was necessary to adapt the sequence to GCC ATT GGA (A2) prior to the fusion to S, resulting in S‐*cph*B‐sA2. Western blot analysis of 25 μg TSP showed that S‐*cph*B‐s produced substantially more CGPase than S‐*cph*B‐sA2 (Figs 1 and 2b) and also showed a higher yield compared to *gpf*‐*cph*B‐s (Fig. 2b). To determine a possible effect of A2 on protein folding, we conducted *in silico* analysis of S‐CPHB‐S and S‐CPHB‐A2 and found different potential α‐helices between AA 4‐25, caused by the integration of Ala‐Ser‐Ser (Fig. S1). This might have an effect on protein stability or folding of the transit peptide. In addition to the expected band at 29 kDa, a band at approximately 35 kDa was detected, corresponding to the unprocessed proteins (Fig. 2b) for S‐*cph*B‐s and S‐*cph*B‐sA2. The same bands were present in isolated chloroplasts (data not shown).

Putative S‐CPHB‐S dimers of 70 kDa were also visible when analysing higher protein concentrations (data not shown). Expression of S‐*chp*B‐s led to the greatest amount of enzyme detected compared to all other constructs. To determine whether differences between the constructs might be caused by different RNA patterns, we conducted Northern blot analysis.

### RNA analysis of different CGPase variants

**Figure 3:**
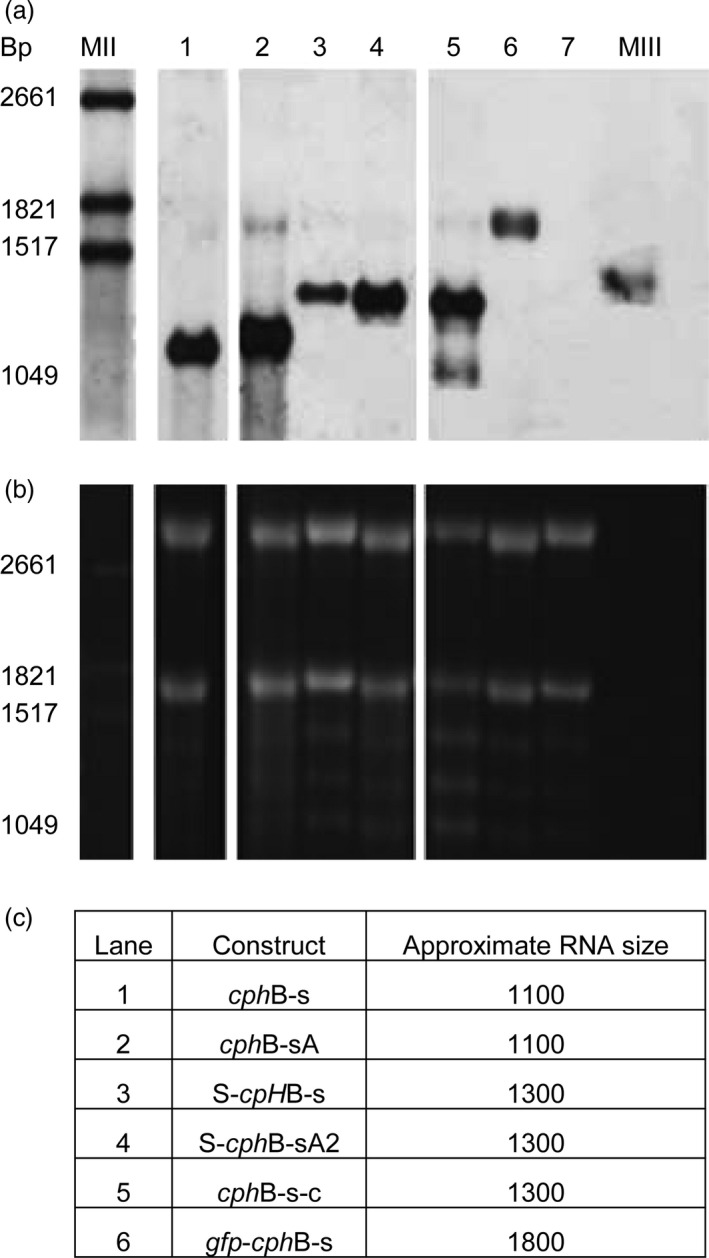
Northern blot (a) and RNA gel loading control (b) of 3 μg RNA isolated from *N. benthamiana* leaves. All samples showed the approximate expected size bands (c) but different signal strength. The loading control (b) shows that samples were loaded equally. MII: RNA marker II with a base pair (bp) range of 1516‐6948 bp, MIII: RNA marker with a bp range of 310‐1517 bp. 1: *cph*B‐s, 2: *cph*B‐sA, 3: S‐*cph*B‐s, 4: S‐*cph*B‐sA2, 5: *cph*B‐s‐c, 6: gfp‐*cph*B‐s, 7: empty vector control.

Northern blot assays were performed to compare the RNA steady‐state levels of the different constructs (Fig. 3a). For all constructs, RNAs corresponding to the calculated size (Fig. 3c) were observed. For *cph*B‐sA, an additional band at approximately 1700 bp was detected. This fragment was weakly detected for *cph*B‐s‐c, which also had a third band at approximately 1.000 bp. While the loading control showed equal amounts of total RNA (Fig. 3b), the strongest signal was observed with *cph*B‐sA. *Cph*B‐s, S‐*cph*B‐sA2, *cph*B‐s‐c and *gpf*‐*cph*B‐s had similar signals. The weakest signals were found for S‐*cph*B‐s. This indicates a possible positive influence of A on transcript stability.

### Activity of *N*‐terminal‐modified CGPase in crude plant extracts

**Figure 4:**
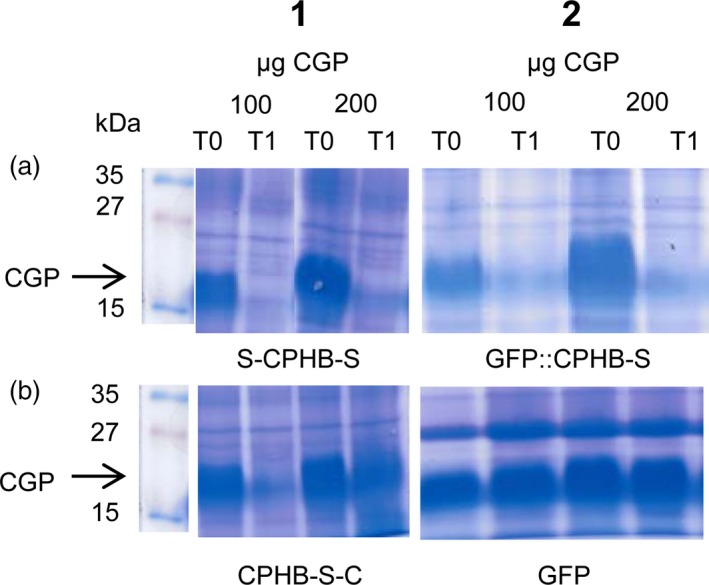
Enzyme activity test in 600 μg TSP crude plant extract. One hundred or 200 μg of cyanophycin was added, and the enzyme reaction was stopped by trichloroacetic acid (TCA) precipitation immediately (T0) or after 12h incubation at room temperature (T1). Total protein was precipitated with TCA and dissolved in 300 μL SDS sample buffer. Then, 30 μL of the sample was loaded on a 12% SDS gel and stained with Coomassie Brilliant Blue for 20 min; kDa: kilodalton; CGP: cyanophycin; 1a: S‐CPHB‐S, 2a: GFP::CPHB‐S, 1b: CPHB‐S‐C, 2b: GFP (green fluorescent protein expressed with vector pICH18711 (Marillonnet *et al*., 2005)) was used as a control.

The activity of the plant‐produced, modified enzymes was determined by adding 100 or 200 μg purified CGP to 600 μg TSP isolated from *N. benthamiana* plants, which were infiltrated with the respective vectors. One reaction was stopped immediately (T0), while the other sample was incubated at room temperature (RT) overnight (T1). Both CGP samples were degraded with proteins from plants infiltrated with S‐*cph*B‐s (Fig. 4:1a) and *gfp*‐*cph*B‐s (Fig. 4:2a). Extracts from plants infiltrated with *cph*B‐s‐c (Fig. 4:1b) led to a nearly complete substrate reduction in the 100 μg CGP sample, while the 200 μg sample of CGP was only partially reduced. No degradation was found after incubation in plant material infiltrated with the GFP‐expressing control vector pICH18711 (Marillonnet *et al*., 2005) (Fig. 4:2b) or with all other constructs used in this work (not shown).

### Feeding CGP‐ and CGPase‐containing pellets to mice results in the absorption of ß‐Asp‐Arg dipeptides

**Figure 5:**
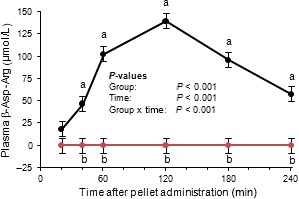
Course of ß‐Asp‐Arg concentrations in mouse plasma after administration of a pellet with cyanophycin only (red) and cyanophycin co‐applied with cyanophycinase (black). LSMEANS ± SE (*n *=* *6/group). Values with different letters (a, b) at the same time points differ between groups (Tukey, *P *< 0.05).

Feeding studies were performed using plant‐made CGP and plant‐made CGPase (S‐CPHB‐S) to investigate the activity of CGPase in the intestine and the bioavailability of the CGP constituent Arg and Asp. The mice were fed protein‐free pellets supplemented with CGP, CGP+CGPase, Asp+Arg or none of these (CON). The pellet mass ingested was comparable between groups (*P *> 0.6) (Table S1). Intake of CGP was similar in mice fed pellets containing CGP or CGP+CGPase (Table S1). Intakes of Arg and Asp in the CGP, CGP+CGPase and Asp+Arg mouse groups were comparable, but intakes were zero in the CON group, as expected. Plasma Asp, ß‐Asp‐Arg, Arg and ornithine Orn concentrations were affected by group, time after administration (with the exception of Orn), and group × time interaction (Table S1). In the plasma of the CGP+CGPase group, we found a relatively large peak, which was shown to be the ß‐Asp‐Arg dipeptide (Figs S2 and 5). This substance did not appear in the plasma of the CGP, Asp+Arg and CON mice, respectively (Fig. 5).

### Arg from ß‐Asp‐Arg dipeptides is not bioavailable in mice

**Figure 6:**
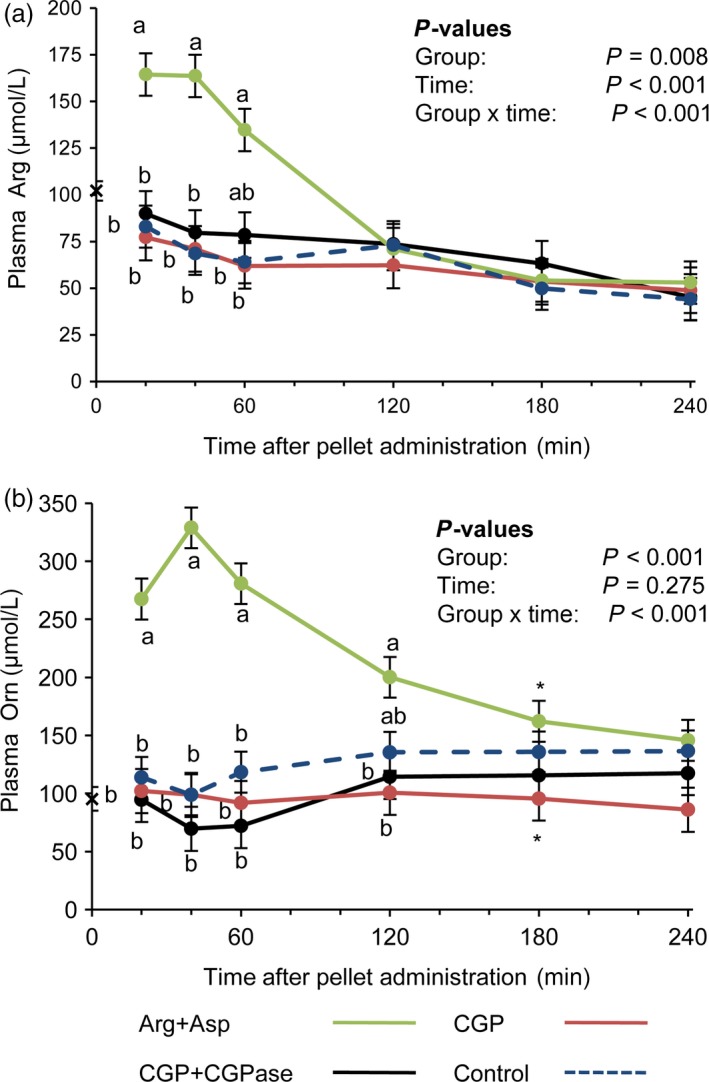
Course of Arg (a) and Orn (b) concentrations in mouse plasma after administration of a pellet with free Asp+Arg (green), cyanophycin only (red), cyanophycin co‐ingested with cyanophycinase (black) and control pellet without supplement (dashed blue). LSMEANS ± SE (*n *=* *6/group). Values with different letters (a, b) at the same time points differ between groups (Tukey, *P *< 0.05). *Values sharing the sign at the same time point tend to differ (Tukey, *P *< 0.10). x: The sign on the *y*‐axis indicates the overall average basal plasma concentration across the four groups.

Free Arg concentrations in the plasma of Asp+Arg mice peaked 20–40 min after pellet intake and decreased thereafter to reach basal levels between 60 and 120 min (*P *< 0.05; Fig. 6a). In contrast, in the CON, CGP and CGP+CGPase groups, the courses of free plasma Arg, Asp (data not shown) and Orn, the product of Arg conversion, were similar and showed no increase (Fig. 6a,b). The course of plasma Orn concentrations in Asp+Arg mice followed the concentration curve of plasma Arg, although the maximal concentration of Orn was approximately twice that of Arg, and baseline was reached again at 180 min (Table S1; Fig. 6b). The pharmacokinetics of the plasma ß‐Asp‐Arg dipeptide in the CGP+CGPase group differed from those of the free plasma Arg in the group fed the pellets with free Asp and Arg (Figs 5, 6). The mean *T*_max_ and *C*_max_ of plasma Arg were 0.5 h and 172 μm, respectively, whereas plasma ß‐Asp‐Arg peaked with a *T*_max_ and *C*_max_ of 1.7 h and 224 μm, respectively (*P *< 0.001 and *P *= 0.115, for *T*_max_ and *C*_max_, respectively). The plasma area under the curve (AUC) was greater for plasma ß‐Asp‐Arg than for free plasma Arg, 527 vs. 282 μm × h (*P *= 0.003), respectively, whereas plasma clearance (CL) for ß‐Asp‐Arg was lower than that for plasma Arg, 7.6 vs. 11.2 L/h (*P *= 0.095), respectively. Among the other proteinogenic AA, only plasma concentrations of glutamic acid (Glu), Ala, isoleucine (Ile) and lysine (Lys) showed a group effect (*P *< 0.05) with higher levels of Glu and Ala in the Asp+Arg group, and higher levels of Lys and Ile in the CON group which received the protein‐free pellet (data not shown).

Group, intestinal location and group × location interaction affected the residual intestinal CGP content 4 h after pellet intake (*P *< 0.05). Co‐administration of CGP+CGPase resulted in a low residual CGP content in the small and large intestine (0.8 and 1.3 μg/mg of dry matter (DM); *P *> 0.1), while in mice fed the pellets with CGP only, relatively high contents of residual CGP were detected in the large intestine (33.4 μg/mg of DM), with smaller amounts of CGP (8 μg/mg) in the small intestine (*P *< 0.05).

## Discussion

### CGPase production in plants

In the present work, we showed for the first time the expression of cyanophycinase in plants and its cyanophycin degrading activity in the gastrointestinal tract (GIT) of mice. We used a high‐yield MagnICON^®^ transient expression system (Marillonnet *et al*., 2004) to determine whether plants can produce an active form of the enzyme cyanophycinase. In contrast to other researchers, who used similar vectors and described a high yield of recombinant protein of approximately 7% (Nausch *et al*., 2012a), 10% (Webster *et al*., 2009) and even 80% of the TSP (Gleba *et al*., 2005), we observed low levels of detectable protein. Because the expected RNA patterns were detected, possible reasons for the low protein yield may be instability of the RNA, low efficiency of translation and protein instability (reviewed by Egelkrout *et al*. (2012) and Ullrich *et al*. (2015)). Improvements in RNA stability and translation can be achieved by adapting the coding region to the codon usage of plants (Barahimipour *et al*., 2015; Perlak *et al*., 1991; Sharp and Li, 1987) and integrating the sequence GCT TCC TCC, which encodes Ala‐Ser‐Ser (A), downstream of the initial start codon (Sawant *et al*., 2001). Accordingly, both steps led to an increase in protein production, but the addition of protease inhibitors was necessary to detect the protein. As already assumed by Sawant *et al*. (2001) the results of this work indicate that the insertion of A led to an increased amount of RNA and also to an increase in protein prior to extraction, while the sensitivity to proteases was not changed.

In contrast to the aforementioned beneficial effect of the insertion of A, the combination of the RuBisCO transit peptide (S) and A2 in the variant S‐CPHB‐A2 substantially reduced protein accumulation compared to S‐CPHB‐S and RNA amounts were not increased, indicating that transcription was not responsible for the difference in protein accumulation. Hence, it appears that the difference is caused by different translational efficiencies or different protein stability *in planta*. *In silico* analysis of the protein indicates an influence in the secondary structure of the transit peptide which might result in aberrant folding and protein degradation. In general, protein targeting to the chloroplast led to high enzyme production. The presence of high amounts of processed protein indicates the successful import of the protein to the chloroplast. In addition, this may indicate that the native protein is protected in the chloroplast because the same protein is unstable in the cytosol. One reason may be the absence of cytosol‐specific proteases in the chloroplast, as previously described for recombinant proteins (Benchabane *et al*., 2008; Pillay *et al*., 2014).

In addition to the processed protein, the unprocessed 35 – kDa protein was detected in the chloroplast fraction. Gils *et al*. (2005), who used the same transit peptide with altered cleavage sites (valine‐cysteine‐Arg and proline‐Ser‐Arg instead of valine‐glutamine‐cysteine in our studies), made a similar observation and suggested that it was due to partially incorrect processing of the target protein. The unprocessed form may either be present in the cytosol or attached to the chloroplast membrane. Assuming that it is located in the cytosol, the *N*‐terminal addition of the signal peptide may protect the protein from degradation as observed for the *N*‐terminal addition of GFP or the *N*‐terminal region of the complete protein.

While the fusion to GFP and S led to an increase in protein accumulation, it was less pronounced for the original *N*‐terminus. The reduced stability of the complete protein was also indicated by the additional bands observed for CPHB‐S‐C, possibly representing degradation products. The *N*‐terminal modification may result in a decreased sensitivity to proteases, as suggested for GFP (Moreau *et al*., 2010; Piron *et al*., 2014), ubiquitin (Hondred *et al*., 1999; Jang *et al*., 2012) and elastin‐like proteins (Floss *et al*., 2008; Patel *et al*., 2007). Nevertheless, we cannot exclude the possibility that the increased levels of GFP::CPHB‐S and S‐CPHB‐S are due to altered translational efficiency.

The increase in protein yield was mirrored by the activity of the protein. S‐CPHB‐S and GFP::CPHB‐S, which had the highest levels of CGPase, showed similar activities. Both enzyme variants decreased the CGP content for both amounts of substrate tested. CPHB‐S‐C, which was produced at lower levels, led to almost complete degradation of 100 μg CGP but only slightly reduced 200 μg CGP. No activity was detected for all other constructs. This indicates that the degradation was independent of the *N*‐terminal modifications but depended on the amount of protein. This is consistent with the observations that the fusion to GFP did not impair biological function of an antigen (Piron *et al*., 2014) and phytochrome B (Yamaguchi *et al*., 1999).

### Absorption of CGP‐derived ß‐Asp‐Arg dipeptides in mice

We report in this study for the first time that co‐application of CGP and CGPase, both isolated from plants, results in the enzymatic breakdown of CGP into dipeptides in the murine GIT, as shown by the increase in β‐Asp‐Arg concentrations in plasma. Upon luminal cleavage of CGP into β‐Asp‐Arg dipeptides by CGPase in the GIT, ß‐Asp‐Arg is apparently absorbed by peptide transporters (Klang *et al*., 2005; Rubio‐Aliaga and Daniel, 2008). Although the *T*_max_ of β‐Asp‐Arg dipeptide occurred at 1.7 h after intake, increased plasma concentration of the constituent Arg or Asp was not detected. ß‐Asp‐Arg may be partly degraded by peptidases in the intestinal epithelium, liberating Asp and Arg. However, because Arg is degraded by arginase to form Orn, urea and, to a lesser degree, nitric oxide and polyamine in the GIT, detectable amounts of Arg do not enter the systemic circulation (Wu *et al*., 2009). As we did not observe an increase in plasma Orn concentrations in the CGP+CGPase group, we conclude that Arg cannot be liberated from β‐Asp‐Arg dipeptides. This is likely because β‐Asp‐Arg dipeptides contain an unusual bond between the C1 amino group of Arg and the C4 carboxy group of Asp. This rare phenomenon was also described for β‐Ala‐(Met)‐His and Gly‐Gly (Matthews and Adibi, 1976). Hence, the lack of an Arg and Orn increase in the plasma indicates that mice do not possess a suitable peptidase to degrade ß‐Asp‐Arg dipeptides. It has been shown that isoaspartyl dipeptidases have different activities in different species (Hejazi *et al*., 2002). Consequently, β‐Asp‐Arg accumulates and is eventually transferred to the blood. The increased plasma ß‐Asp‐Arg concentrations from 40 to 240 min after pellet consumption in the CGP+CGPase group may be explained by two reasons: CGPase was stable at the pH in the small intestine and thus was active for 120 min after administration, after which plasma ß‐Asp‐Arg concentrations started to decline. Alternatively, upon entering the enterocytes, ß‐Asp‐Arg dipeptides accumulated but were only slowly released to the circulation and subsequently detoxified and excreted via urine, which we did not analyse.

Furthermore, the higher Glu and Ala plasma concentrations observed in the Asp+Arg group indicates the interconversion of Arg (via Orn) and Asp to Glu, whereas Asp is also biochemically related to Ala. The higher plasma levels of Lys and Ile in the CON group fed the protein‐free pellet suggest cellular proteolysis and AA efflux to the plasma as a consequence of the lack of a suitable AA pattern necessary for protein synthesis.

The AUC in the Asp+Arg compared to the CGP+CGPase groups suggests that although equimolar amounts of Arg were consumed, a large portion of the free Arg was degraded in the small intestinal tissues to form Orn, as shown by the substantial increase in plasma Orn concentrations. Others have shown that supplemental Arg is rapidly catabolized to Orn by arginase present in the hepatocytes and also in plasma (Wu *et al*., 2009). Judged by the timing of the increase in plasma ß‐Asp‐Arg concentration compared to free Arg (1.7 vs. 0.5 h), we hypothesized that the major site of CGP degradation is the small intestine and not the stomach. This is further supported by the comparatively low residual CGP contents in the small intestine when mice were co‐administered CGP and CGPase, which additionally indicates that CGP was not completely degraded by CGPase within 4 h. When mice were fed pellets with CGP only, CGP levels were high in the large intestine, suggesting that CGP was resistant to colonic fermentation, although bacteria expressing CGPase have been reported in the caecum microbiota of rabbits, sheep and carp (Sallam and Steinbuchel, 2009a).

In conclusion, we showed that plants are able to produce high amounts of active CGPase. Differences in enzyme activity were caused by different CGPase accumulation, indicating that the successful CGP degradation by CGPase depends on the enzyme amount. The greatest accumulation was observed for S‐CPHB‐S and GFP::CPHB‐S; therefore, these two variants are suitable for further investigations related to the production of CGP in plants. The results obtained in the mouse study suggest that plant‐derived CGPase, when co‐ingested with CGP, is active in the mammalian intestine and hydrolyses CGP to form ß‐Asp‐Arg dipeptides, which can be absorbed. However, Arg from these dipeptides is not bioavailable owing to the lack of a suitable dipeptidase. This problem might be solved by the co‐expression of a suitable dipeptidase in combination with CGP and CGPase in plants.

## Experimental procedures

### Construction of transient plant expression vectors

For transient expression, we used the MagnICON^®^ vectors pICH29912 (cytosolic expression) (Marillonnet *et al*., 2005) and pICH26201 for chloroplast‐targeted expression (Fig. 1), which were kindly provided by Nomad Bioscience (Halle/Saale, Germany). The *cph*B coding fragments were integrated using the *Bsa*I cloning site. Vector pet22b*cph*B, carrying a 55 AA *N*‐terminal truncated coding region of the *cph*B_tlr2169_ gene from *Thermosynechococcus elongatus* BP‐1 (UniProt Accession No. P0C8P3) with a *C*‐terminal 6xHistag, was provided by Prof. Dr. Wolfgang Lockau (Institute of Biology‐Plant Biochemistry of the Humboldt‐University of Berlin, Germany). This sequence, called *cph*B‐b, was adapted to the codon usage of *N. tabacum* (Eurofins MWG Operon, Ebersberg, Germany), resulting in *cph*B‐s. The GCT TCC TCC sequence encoding Ala‐Ser‐Ser (A) was integrated downstream of the start codon to improve the efficiency of translation (Sawant *et al*., 2001), using primer *Bsa*I‐*cph*B‐sA‐fw (Table S2), resulting in *cph*B‐sA. *N*‐terminal modifications were added to the coding regions by cloning the corresponding PCR fragments. The sequence of green fluorescent protein (GFP) was amplified from vector pICH18711 (Marillonnet *et al*., 2005), flanked by *Bsa*I (5′) and *Bam*HI (3′) and subcloned into pJet (CloneJET PCR cloning kit, Thermo Scientific, Bonn, Germany). *Cph*B‐s was also flanked by *Bsa*I (3′) and *Bam*HI (5′) and integrated into pJet. Subsequently, *gfp* was fused to *cph*B‐s using the *Bam*HI sites. To create *cph*B‐s‐c, the unmodified coding region as described in the database, a synthetic sequence corresponding to the first 70 AA of the full‐length *cph*B sequence (*cph*B‐s‐c‐pI), was adapted to the codon usage of *Nicotiana tabacum* (Eurofins MWG Operon, Ebersberg, Germany) and cloned into vector pEXA2‐*cph*B‐s‐pI. The sequence coding for AA 71‐330 (*cph*B‐s‐c‐pII) was amplified from *cph*B‐s and flanked by *Bgl*II and *Sal*I restriction sides. This fragment was integrated into pEXA2‐*cph*B‐s‐c‐pI, resulting in the vector pEXA2‐*cph*B‐s‐c. S‐*cph*B‐s was constructed via integration of the coding region *cph*B‐s into pICH26201. For vector pS‐c*cph*B‐sA2, the Ala‐Ser‐Ser AA sequence at position +4 to +12 was adapted from GCT TCC TCC to GCC ATT GGA using primer *cph*B‐sA2‐BsaI‐fw. For propagation, all constructed vectors were transformed into *E. coli* TG1 and validated by sequencing (Eurofins MWG Operon, Ebersberg, Germany). For plant infiltration, the vectors were transformed into *A. tumefaciens* strain ICF320 (Bendandi *et al*., 2010).

### Agroinfiltration of *Nicotiana benthamiana* plants

Transient expression in *N. benthamiana* plants was carried out as described by Nausch *et al*. (2012b) (cultivation and growth of bacteria and plants) and Leuzinger *et al*. (2013) (infiltration process) with modifications (for details see Data S1). Plants were infiltrated using vacuum infiltration with a freeze dryer (Alpha 1‐4 Freeze Dryer, Christ, Germany) at 100 mBar for 2 min. Noninfiltrated leaves were removed, and plants were incubated in the dark overnight before they were returned to their regular growth conditions with a 16h day and 8h night cycle at 20–22 °C.

### Sample preparation and analyses of protein content and RNA

Samples were taken 7, 8 and 9 days post infiltration (dpi), frozen immediately with liquid N_2_ and stored at −80 °C. All leaves were harvested and pooled. Two to three plants per day and constructs were analysed as independent replications. TSP was measured according to Bradford (1976) using Pierce reagent and bovine serum albumin (BSA) (Thermo Scientific) as the standard. Isolation and preparation of total RNA were conducted as described previously (Nausch *et al*., 2012b), and Northern blot analysis was performed as described in Data S1 using the primers *Bsa*I‐*cph*B‐b‐fw, *cph*B‐b‐*Bsa*I‐rv, *cph*B‐s‐*Bsa*I‐rv and *cph*B‐s‐N‐fw (Table S2).

### Western blot analysis

Sample preparation and Western blot analysis were carried out as described by Nausch *et al*. (2012b) with modifications (for details see Data S1). CPHB‐B isolated from *E. coli* was used as a positive control. The primary anti‐CPHB antibody was produced in two Zika rabbits. Serum was obtained via centrifugation of the collected blood, and 0.04% sodium azide was added for storage. A commercial secondary antibody was used (goat anti‐rabbit POD, Dianova, Hamburg Germany), and signals were detected using the ECL chemiluminescence system.

### Analysis of enzyme activity in crude plant extracts

The pooled leaf sample was mixed with chilled phosphate‐buffered saline (PBS) and homogenized using a Polytron (Pt‐MR 2100, Kinematica AG, Switzerland) at maximum speed. TSP (600 μg) was incubated with 100 and 200 μg CGP. The reaction was neutralized (Law *et al*., 2009). Two samples per construct and CGP concentration were analysed. The reaction was stopped immediately or overnight using trichloroacetic acid precipitation. After centrifugation, the pellet was resolved in 300 μL 1× SDS sample buffer (Nausch *et al*., 2012b), and 30 μL of the sample was analysed by 12% SDS‐PAGE. The gel was stained with Coomassie Brilliant Blue R250 (Carl Roth GmbH, Germany) for 20 min.

### Isolation of CGP and CGPase and prediction of protein properties

CGP was isolated from *Solanum tuberosum* tubers (PsbY‐cphA_*TE*_‐12) as described by Neubauer *et al*. (2012), and 10 mg was dissolved in 1 mL 0.1 m HCL pH < 2. CGPase was isolated from *E. coli* BL21 cells carrying pET22b‐*cph*B‐his and *N. benthamiana* leaves using Ni^2+^ NTA affinity chromatography as described in detail in the supplementary information (Data S1). The molecular weight of the proteins was predicted using the sequence manipulation suite home page (Stothard, 2000). Prediction of *in silico* protein folding was carried out using the Phyre2 server (Kelley and Sternberg, 2009; Kelley *et al*., 2015).

### Mouse study and plasma amino acid analysis

The procedures performed in this study were in accordance with the German animal protection regulations and were approved by the relevant authorities (Landesamt für Landwirtschaft, Lebensmittelsicherheit und Fischerei, Mecklenburg‐Vorpommern, Germany; permission No. 7221.3‐1‐017/14).

Male mice (age 49 days) of an unselected control strain (FZTDu; Dietl *et al*. (2004)) bred at the Leibniz Institute for Farm Animal Biology (FBN) in Dummerstorf were housed individually with sawdust bedding at 22 °C and a 12:12‐h dark:light cycle. Mice were fed a standard rodent diet *ad libitum* (Altromin 1314, Altromin GmbH & Co. KG, Lage, Germany; 22.5% crude protein, 5% crude fat, 12.5 MJ ME/kg diet) and had free access to water. Balanced for litter and body weight (BW), the mice were randomly divided into four groups (*n *=* *7/group) according to the type of test pellet fed: cyanophycin (CGP), cyanophycin + cyanophycinase (CGP+CGPase), free L‐Arg and L‐Asp (Asp+Arg) and control (CON).

Protein‐free test pellets were based on 220 mg of a 1:1 mixture of corn starch (Backfee, OsnaNährmittel GmbH, Osnabrück, Germany) and powdered sucrose (Nordzucker AG, Braunschweig, Germany). The basal pellet mixture was supplemented with 30 mg CGP (from *S. tuberosum* tubers), 30 mg CGP + 10 mg CGPase (from *N. benthamiana* leaves), or 15 mg Arg (Degussa, Frankfurt/Main, Germany) plus 15 mg Asp (Reanal, Budapest, Hungary). This corresponded to 1 mg CGP, 0.33 mg CGPase, or 0.5 mg Arg or Asp, respectively, per g BW. The pellet for the control group (CON) contained none of these supplements. Dry matter was adjusted to 250 mg for each pellet with the starch/sucrose mixture to achieve comparable energy contents. A volume of 40–45 μL of pH 3 water was added to the mixtures, the mixture was filled in 1‐mL plastic syringes (Omnifix^®^ 40 Solo, Braun, Melsungen, Germany), and pellets were pressed manually with a plunger. Pressed pellets were stored overnight at 4 °C before consumption.

At the age of 79–92 days, the mice were transferred to cages without sawdust after overnight food withdrawal. Test pellets were offered to the mice for 15 min, the remaining food was collected, and the animals were transferred back to their original cages. The tips of their tails were snipped to collect blood in sodium‐heparinized microhematocrit capillary tubes (Marienfeld, Lauda‐Königshofen, Germany) at 0, 20, 40, 60, 120, 180 and 240 min after pellet administration. The capillaries were immediately put on ice and were centrifuged for 3 min at 10 000 × ***g*** and 4 °C. The isolated plasma was diluted with ultrapure water and stored at −20 °C. Immediately after the 240 min, blood samples were taken, mice were killed by cervical dislocation. The abdomen was opened, the small and large intestine were isolated, and the total contents were rinsed with 3 mL cold PBS, weighed, and stored frozen at −20 °C The residual CGP concentration of the small and large intestine in the CGP, CGP+CGPase and free Asp+Arg mice were quantified as described in Data S1. We used only mice with >40% intake of their respective pellets. Thus, valid observations were obtained for 6, 6, 7 and 7 mice in the CGP, CGP+CGPase, Asp+Arg and CON groups, respectively. Plasma AA were analysed by HPLC separation with fluorescence detection of o‐phthaldialdehyde derivatives on a 250 × 4 mm HyperClone ODS (C18) 120 Å (Phenomenex, Aschaffenburg, Germany) as described (Kuhla *et al*., 2010). Standard AA (A9906 Sigma, Munich, Germany) allowed assignment of retention times and quantification. The β‐Asp‐Arg dipeptide, eluting at a retention time of 3.8 min, was identified using isolated CGP degraded by CGPase (Fig. S2). Enzyme hydrolysis of CGP (200 μg) was performed as described (Law *et al*., 2009) with minor modifications. CGP (10 mg/mL in 0.1 m HCL) was diluted in PBS (pH 7.2), and 50 μg of S‐CPHB‐S (1 mg/mL) isolated from *N. benthamiana* was added. Samples were incubated at RT overnight. CGPase was removed from the hydrolysis mixture by centrifugation with Roti^®^‐Spin MINI‐3 cartridges (3 kDa, Carl Roth GmbH + Co. KG, Karlsruhe, Germany) for 20 min at 17 000 × ***g*** and 4 °C. The β‐Asp‐Arg dipeptide was quantified by the AUC using a four‐point calibration. The detection limit was 0.5 μm. An aliquot of the β‐Asp‐Arg dipeptide filtrate (100 μL) was hydrolysed in 1 mL of 6 N HCl at 110 °C for 22 h with ascorbic acid as an antioxidant under a N_2_ atmosphere to confirm the Asp and Arg constituents only. After HCl removal under a N_2_ stream at 60 °C, the residue was diluted in 1 mL H_2_O, centrifuged for 20 min at 17 000 × ***g*** and 4 °C and analysed for AA concentrations. Based on the concentrations of Arg and Asp, the amount of β‐Asp‐Arg was recalculated. Then, plasma concentrations of Asp, Arg, β‐Asp‐Arg dimer and Orn were quantified. For calculation of the pharmacokinetics of these metabolites, data were normalized for the pellet intake and BW after subtraction of the mean concentrations of the corresponding AA of the CON group. The AUC for the β‐Asp‐Arg dimer and Arg between 0 and 4 h after administration was calculated with TableCurve 2D V 5.01 software (SYSTAT Software Inc., Chicago, IL). The time (*T*_max_) at which the maximal plasma concentration, *C*_max_, was reached was computed using a best curve fit. Plasma clearance was calculated from the administered dose of CGP (converted to β‐Asp‐Arg equivalents) or Arg (μmol) divided by the AUC.

### Statistical analysis of mouse study

Mouse data were evaluated with SAS 9.4 software (SAS Institute Inc. 2011, Cary, NC). A repeated‐measures ANOVA implemented in a mixed model was used with the fixed effects of group (CGP+CGPase, CGP, Asp‐Arg, CON) and time of blood sampling (20, 40, 60, 120, 180, and 240 min), as well as the interaction term. The effect of repeatedly sampled animals was considered random. The covariance structure was autoregressive (AR1). For singly measured variables, a one‐way ANOVA was used with group as a fixed effect. Residual intestinal CGP contents were analysed by a two‐factorial ANOVA with group and intestinal location (small and large intestine) as fixed factors and the interaction term. Pellet intake did not differ among groups and was not considered a factor. Effects were considered significant at *P *≤ 0.05, and group differences were tested using the Tukey–Kramer test. Significance levels of *P *< 0.10 were considered a statistical trend. Data are presented as least‐square means (LSMEANS) and their standard errors (SE).

## Conflict of interest

The authors declare no conflict of interest.

## Supporting information


**Figure S1.** *In silico* secondary structure determination of S‐CPHB‐S and S‐CPHB‐A2 was carried out using the Phyre2 server (Kelley and Sternberg, 2009; Kelley *et al*., 2015).
**Figure S2.** Chromatogram of the ß‐Asp‐Arg dipeptide after enzymatic degradation of cyanophycin with cyanophycinase and subsequent enzyme removal (blue); Asp and Arg signals after acid hydrolysis of the ß‐Asp‐Arg dipeptide (red); LU, luminescence units.

**Table S1**. Body weight, intakes of pellets, cyanophycin (CPG), cyanophycinase (CPGase), and free Asp and Arg, as well as concentrations of plasma Asp, ß‐Asp‐Arg dipeptide, Arg, and Orn in four groups of mice fed protein‐free pellets supplemented with CPG, CPG+CPGnase, Asp+Arg, or none of these (control; CON).
**Table S2**. Sequence of primers used in this study^1^

**Data S1.** Detailed experimental procedure.
