# Supplementary material for: Stable production of cyanophycinase in Nicotiana benthamiana and its functionality to hydrolyse cyanophycin in the murine intestine
Source: Plant Biotechnol J. 2016 Dec 18;15(5):605–13. doi: 10.1111/pbi.12658 (PMC5399006; doi:10.1111/pbi.12658)
Supplement: Supplementary file 1 — Figure S1. In silico secondary structure determination of S‐CPHB‐S and S‐CPHB‐A2 was carried out using the Phyre2 server (Kelley and Sternberg, 2009; Kelley et al., 2015). Figure S2. Chromatogram of the ß‐Asp‐Arg dipeptide after enzymatic degradation of cyanophycin with cyanophycinase and subsequent enzyme removal (blue); Asp and Arg signals after acid hydrolysis of the ß‐Asp‐Arg dipeptide (red); LU, luminescence units. [file PBI-15-605-s002.docx]

**Figure S1**


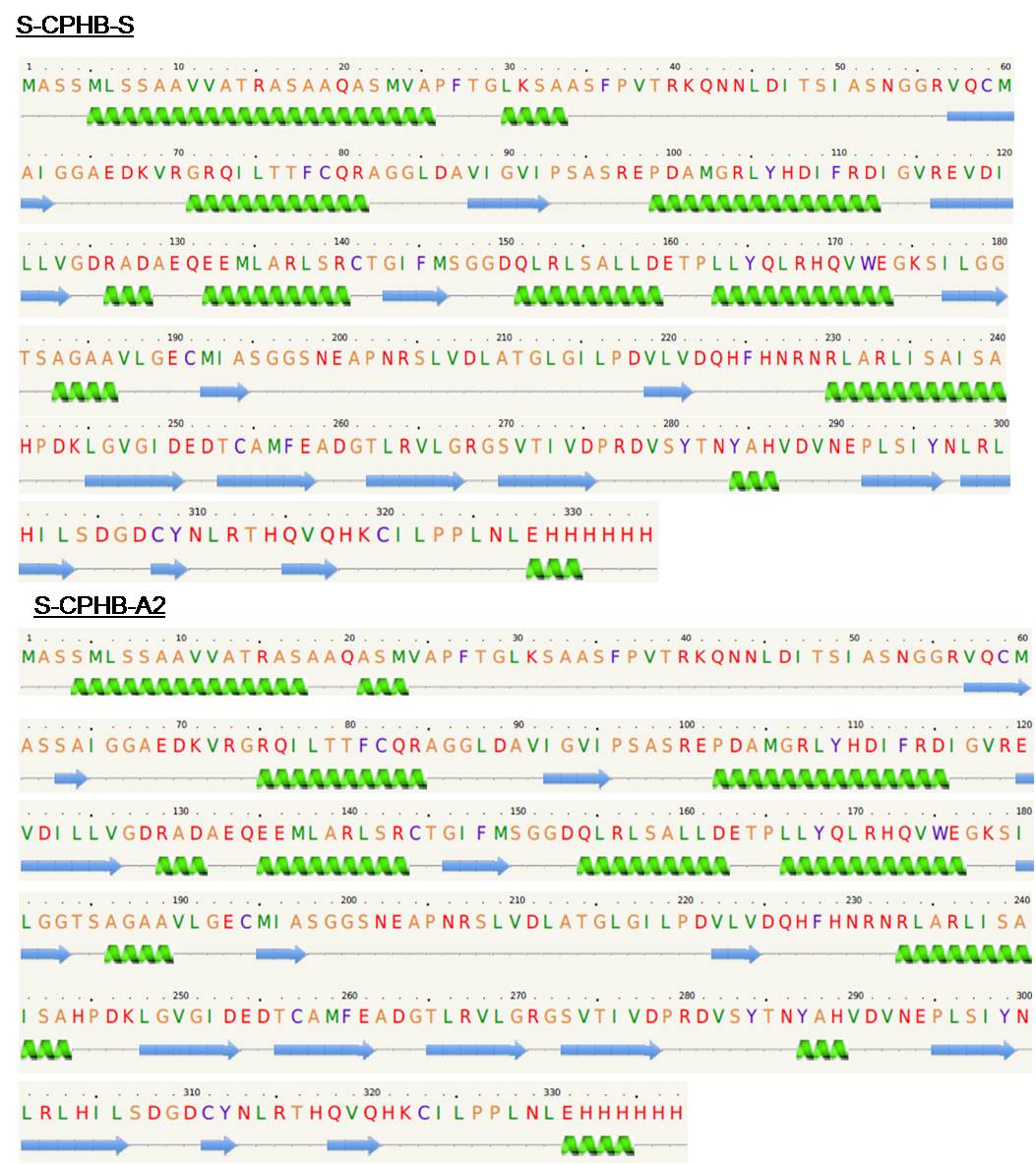


Fig. S1: *In silico* secondary structure determination of S-CPHB-S and S-CPHB-A2 was carried out using the Phyre2 server (Kelley *et al.* 2009, Kelley *et al.* 2015).

**Figure S2**

Fig. S2: Chromatogram of the ß-Asp-Arg dipeptide after enzymatic degradation of cyanophycin with cyanophycinase and subsequent enzyme removal (blue); Asp and Arg signals after acid hydrolysis of the ß-Asp-Arg dipeptide (red); LU, luminescence units.
