# Supplementary material for: Stable production of cyanophycinase in Nicotiana benthamiana and its functionality to hydrolyse cyanophycin in the murine intestine
Source: Plant Biotechnol J. 2016 Dec 18;15(5):605–13. doi: 10.1111/pbi.12658 (PMC5399006; doi:10.1111/pbi.12658)
Supplement: Supplementary file 2 — Table S1. Body weight, intakes of pellets, cyanophycin (CPG), cyanophycinase (CPGase), and free Asp and Arg, as well as concentrations of plasma Asp, ß‐Asp‐Arg dipeptide, Arg, and Orn in four groups of mice fed protein‐free pellets supplemented with CPG, CPG+CPGnase, Asp+Arg, or none of these (control; CON). Table S2. Sequence of primers used in this study1 [file PBI-15-605-s003.docx]

**Table S1.** Body weight, intakes of pellets, cyanophycin (CPG), cyanophycinase (CPGase), and free Asp and Arg, as well as concentrations of plasma Asp, ß-Asp-Arg dipeptide, Arg, and Orn in four groups of mice fed protein-free pellets supplemented with CPG, CPG+CPGnase, Asp+Arg, or none of these (control; CON).

|  | **CPG** | **CPG+**  **CPGase** | **Asp+**  **Arg** | | **CON** | **SE^1^** | **P≤** | | |
| --- | --- | --- | --- | --- | --- | --- | --- | --- | --- |
|  |  |  |  | |  |  | **G*** | **T** | **G x T** |
| **BW*, g** | 39.4 | 39.5 | 39.0 | | 41.8 | 1.74 | 0.615 | -† | -† |
| **Intakes** |  |  |  | |  |  |  |  |  |
| **Pellet, mg** | 223 | 207 | 210 | | 186 | 20.4 | 0.603 | - | - |
| **CPG, mg ˑ kg^-1^ BW** | 683.5^a^ | 662.2^a^ | 0^b^ | | 0^b^ | 58.89 | 0.001 | - | - |
| **CPGase, mg ˑ kg^-1^ BW** | 0^b^ | 260^a^ | 0^b^ | | 0^b^ | 26.54 | 0.001 | - | - |
| **Asp, mg ˑ kg^-1^ BW** | 290.6^a^‡ | 281.1^a^ | 324.1^a^ | | 0^b^ | 29.75 | 0.001 | - | - |
| **Arg, mg ˑ kg^-1^ BW** | 393.5^a^ | 381.5^a^ | 324.1^a^ | | 0^b^ | 37.5 | 0.001 | - | - |
| **Plasma concentrations, µmol/L**§ | | |  | |  |  |  |  |  |
| **Asp** | 7.3^a^¶ | 10.1^ab^ | 16.3^b^ | 14.0^ab^¶ | | 1.92 | 0.012 | 0.019 | 0.091 |
| **ß-Asp-Arg** | 0^a^ | 77^b^ | 0^a^ | 0^a^ | | 6.5 | 0.001 | 0.001 | 0.001 |
| **Arg** | 62.5^a^ | 71.7^ab^¶ | 106.9^b^¶ | 63.7^a^ | | 9.79 | 0.008 | 0.001 | 0.001 |
| **Orn** | 95.8^b^ | 97.3^b^ | 230.9^a^ | 123.2^b^ | | 14.2 | 0.001 | 0.275 | 0.001 |

Data are shown as LSMEANS ± SE, and *n* = 6 – 7 mice per group.

*G = group, T = time, G x T = group x time interaction, body weight (BW).

†Singly measured variables were analyzed with one-way ANOVA.

‡Values differing among groups have different letters (a, b) on the same line (Tukey, *P* < 0.05).

§Basal plasma Arg, Asp, and Orn concentrations in mice prior to pellet intake were 102 ± 5.1, 12.8 ± 1.5, and 99.5 ± 10.2 µmol ˑ L^-1^, respectively (*P* > 0.5).

¶Values sharing the sign on the same line tend to differ (Tukey, *P* < 0.10).

**Table S2.** Sequence of primers used in this study^1^

| **Name** | **Sequence** | **Vector** |
| --- | --- | --- |
| **BsaI-*cph*B-b-fw** | 5'-TTTT**GGTCTC**ACATGGCGATCGGGGGAGCGGAGG-3' | p*cph*B-b |
| ***cph*B-b-BsaI-rv** | 5'-TTTT**GGTCTC**AAAGCTTAGTGGTGGTGGTGGTGGTGCTCG-3' | p*cph*B-b |
| **BsaI-*cph*B-s-fw** | 5'-TTTT**GGTCTC**ACATGGCCATTGGAGGGGCAG-3' | p*cph*B-s,  pS-*cph*B-s |
| **BsaI-*cph*B-sA-fw** | 5'TTTT**GGTCTCAC**ATGgcttcctccGCCATTGG-3' | p*cph*B-sA |
| ***cph*B-s-BsaI-rv** | 5'-TTTT**GGTCTCAAAGC**TTAGTGATGGTGATGATGATGTTCC-3' | p*cph*B-s(A),  pS-*cph*B-s(A2) |
| **BamHI-*cph*B-s-fw** | 5'-TTTT**GGATCC**ATGGCCATTGGAGGGGCAG-3' | pGFP-*cph*B-s |
| **BsaI-GFP-fw** | 5‘-TTTTGGTCTCACATGGTGAGCAAGGGCGAGGAGCTGTTCACC-3‘ | pGFP-*cph*B-s |
| **GFP-BamHI-rv** | 5‘-CCCCCGATCGCCAT**GGATCC**CTTGTACAGCTCGTCCATGC-3‘ | pGFP-*cph*B-s |
| **BsaI-c*ph*B-s-c-pI-fw** | 5'-TTTTGGATCC**GGTCTC**ACATGTTGTACAGAATTC-3' | p*cph*B-s-c |
| ***cph*B-s-c-pI-SalI-rv** | 5'-TTTT**GTCGAC**GAATATTGTAGGAG-3' | p*cph*B-s-c |
| **BglII-*cph*B-s-c-pII-fw** | 5'-TTTTGC**AGATCT**TGACTACCTTTTGCCAAAGAGC-3' | p*cph*B-s-c |
| ***cph*B-s-c-pII-SalI-BsaI-rv** | 5'-TTTT**GTCGAC**GGATCC**GGTCTC**AAAGCTTAGTGATGGTGATGATGATG-3' | p*cph*B-s-c |
| **BsaI-*cph*B-sA2-fw** | 5'-TTTT**GGTCTC**ACATGgcctctagcGCCATTGG-3' | pS-*cph*B-sA2 |
| ***cph*B-s-N-fw** | 5’TTTTCAGAAGATAAGGTTAGAGGAAGGCAG-3 | Northern |

^1^Restriction sites used in this study are shown in bold. Start and stop codons of *cph*B are underlined. The added amino acids A-S-S are shown in small letters.
