# Supplementary material for: Stable production of cyanophycinase in Nicotiana benthamiana and its functionality to hydrolyse cyanophycin in the murine intestine
Source: Plant Biotechnol J. 2016 Dec 18;15(5):605–13. doi: 10.1111/pbi.12658 (PMC5399006; doi:10.1111/pbi.12658)
Supplement: Supplementary file 3 — Data S1. Detailed experimental procedure. [file PBI-15-605-s001.docx]

**Text S1 Experimental Procedure**

**Agroinfiltration of *N. benthamiana* plants**

Modifications: the volume of infiltration buffer was increased from 50 mL to 4 L. Therefore, 400 mL of an *A. tumefaciens* secondary overnight culture was grown and treated as described for the primary culture (Nausch *et al*. 2012). The resulting pellet was dissolved in 4 L infiltration buffer (Marillonnet *et al*. 2004)), and 1 mL of the detergent Silwet Gold (Spieß-Urania; Hamburg, Germany) was added.

**RNA analysis**

A 3 µg aliquot of RNA was separated by gel electrophoresis on a 1.5% agarose gel in MOPS buffer (20 mM MOPS, 5 mM sodium acetate, 1 mM EDTA, 5% formaldehyde, pH 7). Blotting, hybridization and signal detection were performed as described for Southern blot analysis by Nausch *et al.* (2012) with modifications. The membrane was prehybridized for 3 h at 51°C, DIG-labeled probes were amplified using the primers BsaI-*cph*B-b-fw, *cph*B-b-BsaI-rv, *cph*B-s-BsaI-rv and *cph*B-s-N-fw (Table S2) and the membrane was hybridized to the probe at 51°C.

**Western blot**

Modifications during sample preparation: PMSF in the protein extraction buffer was replaced with protease inhibitor cocktail tablets (Complete Tablets, Mini, EASYpack, Roche, Mannheim, Germany) according to the manufacturer’s recommendations. Total soluble protein (TSP) was precipitated using trichloroacetic acid at 4°C for 2.5 h and dissolved in 30 µL 1x sample buffer (10% glycerin, 150 mM Tris (pH 6.8), 3% SDS, 1% mercaptoethanol, and 2.5% bromophenol blue).

After separation, the samples were blotted on a 0.45 µm nitrocellulose Amersham Hybond ECL membrane (GE Healthcare Life Science, Darmstadt, Germany), which was blocked overnight at room temperature (RT) in TBS+T buffer (10 mM Tris, 15 mM NaCl, pH 7.6, 0.05% Tween) containing 5% skim milk powder.

Antibody concentration and incubation: the primary serum was used at a 1:6000 dilution and incubated for 2 h at RT. After 3 washing steps, the secondary antibody (goat anti-rabbit POD, Dianova Hamburg, Germany) was diluted 1:20,000 and incubated for 1 h at RT. After another 3 washing steps, the signals were detected using the ECL chemiluminescence system, and the membrane was exposed to Amersham Hyperfilm^TM^ ECL films (GE Healthcare Limited, Darmstadt Germany) for 2 and 5 min.

**Analysis of enzyme activity in crude plant extracts**

**PBS:** 140 mM NaCl, 10 mM KCl, 6.4 mM Na_2_HPO_4_, 2 mM KH_2_PO_4_, pH 7.2.

**Ni^2+^ NTA affinity purification of cyanophycinase (CPGase)**

A single colony of *E. coli* BL21 carrying pET22b-*cph*B-His (Lockau unpublished) was grown in 50 mL Luria-Bertani (LB) medium (Carl Roth GmbH, Karlsruhe, Germany) containing 1 % glucose, 125 µg^.^ml^-1^ ampicillin at 37°C for 3 h . After centrifugation (10 min, 4,600^.^g, RT), the pellet was dissolved in 100 mL of the same medium and incubated for 2 h at 37°C. Following centrifugation (10 min, 4,600 g, RT), the pellet was dissolved in 200 mL LB medium, containing 250 µg^.^ml^-1^ ampicillin and 1 mM IPTG and grown overnight at RT. This culture was centrifuged (15 min, 4,600 g, RT), and the pellet was dissolved in 30 mL NPI buffer (50 mM NaH_2_PO_4_·H_2_O, 300 mM NaCl, pH 8) containing 10 mM imidazole. Cells were lysed using an ultrasonic unit on ice (2x30 s, output 120) and subsequently centrifuged for 15 min at 4°C, 4,600 g. The supernatant was transferred to a new tube and centrifuged again for 1 h, 4°C, 12,000 g. A 2.7 mL ProBond®Resin (Novex® Life Technologies™, Van Allen Way Carlsbad, CA, USA,) Ni^2+^-NTA matrix was equilibrated with 25 mL NPI buffer. Then, 20 ml of supernatant was loaded on the column, which then was washed twice with 25 mL NPI buffer containing 20 and 30 mM imidazole. Protein was eluted from the matrix surface using 6 fractions of NPI buffer containing 300 mM imidazole. Fractions 2, 3 and 4 were desalted using a PD-10 Sephadex® G-25M column (Pharmacia Biotechnology, Uppsala, Sweden), which was equilibrated with 50 mL PBS (pH 7.5) and eluted with 3.5 mL of the same buffer. Aliquots of 200 µL were stored at -20°C or 4°C for next day use. CPGase was isolated from N. benthamiana leaves by homogenization of leaf material from one whole plant with a Polytron (2x30 s, 19,000 rpm) in cold 50 mM Na_2_HPO_4_·H_2_O buffer (pH 8) containing 0.3 M sucrose and 0.24 M NaCl. Homogenized material was filtered through 3 layers of Miracloth (Merck Millipore, Germany) and centrifuged (4°C, 45 min, 12,000 g). CPHB was isolated as described above, but 15 mL column matrix was used. Therefore, the amounts of buffers were adjusted, and all fractions were rebuffered with PBS (pH 7.5) using Vivaspin 20 concentration tubes according to the manufacturer’s protocol (Sigma Aldrich, Steinheim, Germany).

**CGP quantification in the large and small intestine**

Intestinal digesta samples were stored at -20°C. Cyanophycin (CGP) analysis was conducted as described by Hühns *et al*. (2008) with modifications. Freeze-dried intestinal digesta samples (20-35 mg) were analyzed. Soluble proteins were extracted with 1 mL 50 mM Tris (pH 8) for 30 min in a shaker (1,000 rpm). After a centrifugation step (13,200 rpm, RT, 15 min), the supernatant was discarded and the pellet resuspended in 1 mL of 0.1 M HCl. After another centrifugation step, 800 µL of the supernatant was collected and used for CGP analysis. Tubes with 1 to 10 µL of sample were filled with 0.1 M HCl to a final volume of 800 µL. After addition of 200 µL of 5 x RotiQuant Bradford reagent (Cat. No. K015.1, Carl Roth GmbH + Co. KG, Karlsruhe, Germany) and a 5 min incubation, samples were measured at 595 nm in microcuvettes (Cat. No.: 67.742; Sarstedt AG & Co., Nümbrecht, Germany). A calibration curve was prepared with purified CGP from tobacco leaves or potato tubers, extracted as described by Neubauer (2012), with a concentration range of 1 to 5 µg ⋅ mL^-1^ CGP. OD values of the control mice were subtracted from OD values of samples from the CGP and CGP+CGPase groups.
